# Bacterial Survival under Extreme UV Radiation: A Comparative Proteomics Study of *Rhodobacter* sp., Isolated from High Altitude Wetlands in Chile

**DOI:** 10.3389/fmicb.2017.01173

**Published:** 2017-06-26

**Authors:** Vilma Pérez, Martha Hengst, Lenka Kurte, Cristina Dorador, Wade H. Jeffrey, Ruddy Wattiez, Veronica Molina, Sabine Matallana-Surget

**Affiliations:** ^1^Laboratory of Molecular Ecology and Applied Microbiology, Department of Pharmaceutical Sciences, Universidad Católica del NorteAntofagasta, Chile; ^2^Centre for Biotechnology and BioengineeringSantiago, Chile; ^3^Programa de Doctorado en Ciencias Biológicas, Facultad de Ciencias de la Salud, Universidad de AntofagastaAntofagasta, Chile; ^4^Laboratory of Microbial Complexity and Functional Ecology, Institute of Antofagasta and Department of Biotechnology, Universidad de AntofagastaAntofagasta, Chile; ^5^Center for Environmental Diagnostics and Bioremediation, University of West Florida, PensacolaFL, United States; ^6^Proteomics and Microbiology Laboratory, Research Institute of Biosciences, University of MonsMons, Belgium; ^7^Department of Biology, Faculty of Natural and Exact Sciences, Universidad de Playa AnchaValparaíso, Chile; ^8^Division of Biological and Environmental Sciences, Faculty of Natural Sciences, University of StirlingStirling, United Kingdom

**Keywords:** extreme environment, proteomics, UV radiation, Chilean Altiplano, osmoprotectants

## Abstract

Salar de Huasco, defined as a polyextreme environment, is a high altitude saline wetland in the Chilean Altiplano (3800 m.a.s.l.), permanently exposed to the highest solar radiation doses registered in the world. We present here the first comparative proteomics study of a photoheterotrophic bacterium, *Rhodobacter* sp., isolated from this remote and hostile habitat. We developed an innovative experimental approach using different sources of radiation (*in situ* sunlight and UVB lamps), cut-off filters (Mylar, Lee filters) and a high-throughput, label-free quantitative proteomics method to comprehensively analyze the effect of seven spectral bands on protein regulation. A hierarchical cluster analysis of 40 common proteins revealed that all conditions containing the most damaging UVB radiation induced similar pattern of protein regulation compared with UVA and visible light spectral bands. Moreover, it appeared that the cellular adaptation of *Rhodobacter* sp. to osmotic stress encountered in the hypersaline environment from which it was originally isolated, might further a higher resistance to damaging UV radiation. Indeed, proteins involved in the synthesis and transport of key osmoprotectants, such as glycine betaine and inositol, were found in very high abundance under UV radiation compared to the dark control, suggesting the function of osmolytes as efficient reactive oxygen scavengers. Our study also revealed a RecA-independent response and a tightly regulated network of protein quality control involving proteases and chaperones to selectively degrade misfolded and/or damaged proteins.

## Introduction

The Andean plateau in Chile is exposed to extreme UV radiation doses as a consequence of high elevation and lower total ozone column (TOC) levels (Cordero et al., 2016). Salar de Huasco (3800 m.a.s.l) is a high altitude saline wetland in Chile, presenting poly-extreme environmental conditions such as negative water balance, broad range of salinity (0.29 to 49.1 PSU; Hernández et al., 2016), large daily thermal amplitude (-10 to +25°C; Risacher et al., 2003) and one of the highest solar radiations registered in the world (over 1000 Wm^-2^; Cabrol et al., 2014; Cordero et al., 2016; Hernández et al., 2016; Molina et al., 2016). The study of model bacteria inhabiting those hostile and remote environments is of great interest (i) to better predict the impact of future climate change, (ii) in astrobiology to understand the origin and early evolution of life on Earth, and (iii) for many potential applications in biotechnological processes (Cockell et al., 2000; Cavicchioli et al., 2002; Zepp et al., 2011).

By the end of the 21st century, it is predicted that UVB radiation at the surface of the Earth will have risen by approximately 5–10% in temperate latitudes and by 20% in high latitudes (Zepp et al., 2011; Häder et al., 2015). UV radiation is one of the most detrimental abiotic factors impacting microorganisms at both community and cellular levels, affecting the microbial diversity and dynamics of community structure, as well as causing damage to important biomolecules such as lipids, DNA and proteins (Matallana-Surget et al., 2008; Matallana-Surget and Wattiez, 2013; Santos et al., 2013; Ayala et al., 2014; Pavlopoulou et al., 2016). Bacteria, especially extremophiles inhabiting harsh environments permanently exposed to damaging solar radiation, have evolved different strategies to cope with UV stress, mainly relying on efficient DNA repair mechanisms and/or active defense against UV-induced oxidative stress, and consequently need to tightly regulate their proteome (Chen et al., 2009; Matallana-Surget et al., 2009, 2014; Gao and Garcia-Pichel, 2011; Albarracín et al., 2012; Matallana-Surget and Wattiez, 2013).

Several studies have reported significant variations in the response to UV radiation of several bacteria from aquatic enviroments (Sommaruga, 2001; Fernández Zenoff et al., 2006; Joux et al., 2009; Matallana-Surget et al., 2012b), suggesting that a repercussion of solar radiation on the ecosystem leads to altered community structure that may determine the resilience of ecological systems to changing conditions. It has been demonstrated that in high altitude environments such as alpine lakes (Sommaruga et al., 1997; Pérez and Sommaruga, 2007) and high-altitude Andean lakes (Fernández Zenoff et al., 2006; Farías et al., 2009), UV radiation alters community composition, bacterial growth efficiency and productivity. Only a very few studies (Hernández et al., 2016; Molina et al., 2016) have used high altitude solar radiation to assess the response of natural microbial communities, and only one study has focused on a bacterial model, *Acinetobacter* strain isolated from Lake Verde at the Argentinian Andean Puna (4400 m.a.s.l), to analyze the response in terms of protein regulation (Kurth et al., 2015). This recent study on *Acinetobacter* sp. V3 using UVB lamps and a gel-based proteomics approach showed an up-regulation of catalase and proteins associated with amino acid and protein synthesis, and down-regulation of proteins involved in glycolysis, fatty acid cycle and electron respiratory chain (Kurth et al., 2015).

Culture-independent studies undertaken in Salar de Huasco using 16S clone rRNA library analysis of aquatic and sediment samples (Dorador et al., 2008, 2010, 2013) indicated that this environment possesses highly endemic diverse communities, with species belonging to Proteobacteria, Bacteroidetes, Verrumicrobia and Firmicutes. Phylotypes belonging to the *Rhodobacteraceae* family were found to be highly abundant, in which *Rhodobacter* species were found to be one of the most representative genus. In this context, a *Rhodobacter* sp., isolated from microbial mats in Salar de Huasco was chosen as a bacterial model to study its response and adaptation to UV radiation. *Rhodobacter* species are versatile purple non-sulfur photoheterotrophic bacteria capable of anoxygenic photosynthesis as well as aerobic and anaerobic respiration, according to the encountered environmental conditions (Imhoff et al., 1984; Imam et al., 2013). Moreover, we have sequenced the entire genome of this bacterium (Pérez et al., unpublished), thus facilitating proteomics studies.

In our study, we developed a challenging and innovative experimental design to comprehensively study the protein regulation of *Rhodobacter* sp. in response to both natural sunlight and UVB lamps, using high-throughput quantitative proteomics. To the best of our knowledge, this is the first proteomics study to assess the response of a bacterium isolated from extreme high altitude wetlands and exposed during Southern Hemisphere spring to high altitude UV radiation in its hostile and isolated ecosystem of origin. Exposure of bacterial cells to natural solar radiation limits any bias generated by the use of an artificial solar simulator, such as spectral and irradiance differences (Sayre et al., 1990). The use of cut-off filters (Mylar, Lee filters) allowed us to expose the bacterial cells to three natural treatments; Full Sun radiation, PAR (Photosynthetically active radiation)+UVA and PAR. By comparing the three different natural light treatments between each other, we were able to analyze the impact of six different spectral bands (Full Sun, PAR, PAR+UVA, UVA+UVB, UVB and UVA). We also compared the effects of natural sunlight with artificial UVB radiation, providing new insights on the molecular adaptation of bacteria to extreme doses of UV radiation, in a natural environment and in laboratory conditions.

## Materials and Methods

### Bacterial Isolation and Growth Conditions

Microbial mat samples from Salar de Huasco, situated in the Tarapaca Region, Chile (20°18′S, 68°50′W) at 3800 m.a.s.l, were inoculated in marine broth (Difco) at 28°C for 14 days and spread-plated onto marine agar (MA, Difco). The isolates were identified according to their 16S rRNA gene sequence. Genomics DNA of the strain was extracted using the PowerSoil^®^ DNA Isolation Kit (Mo Bio) following the manufacter’s instructions. The 16S rRNA gene was amplified by PCR, using universal primers 27F and 1542R (Stackebrandt and Liesack, 1993). The PCR product was sequenced (Macrogen Inc., Korea) and compared with sequences available in the RDP database. For exposure studies, *Rhodobacter* sp. was grown aerobically in 1L of Artificial Sea Water (ASW) with 0.5% NaCl and supplemented with 3 mM D-glucose (ASW-G), vitamins and trace elements (Eguchi et al., 1996) after two pre-cultures on a rotary shaker (120 rpm) at 28°C. At pre-stationary phase, cells were harvested for light treatment exposures by centrifugation at 8000 g for 5 min at room temperature and resuspended in unsupplemented ASW medium with a density of 1x10^7^ cells mL^-1^.

### Field Radiation Experiments

Experiments were conducted during Southern Hemisphere spring in November 2015 at Salar de Huasco. To maintain temperature, samples were incubated in a freshwater stream named H0 (Dorador et al., 2010). The stream had transparent waters and registered a constant temperature of 16°C for the duration of the experiment.

The pre-stationary phase culture of *Rhodobacter* sp. was divided equally for the following four treatments: (i) Full Sun (UVA+UVB+PAR); (ii) PAR+UVA, using Mylar D foil (50% transmittance at 320 nm) to exclude UVB; (iii) PAR, using 209.3 neutral density Lee filter (50% transmittance at 400 nm) to exclude all UV radiation, and (iv) Dark (bags were wrapped with heavy black plastic). From these four treatments, specific data could also be inferred for UVB (difference between full sun and mylar treatment), UVA + UVB (difference between full sun and Lee filter treatments), and UVA (difference between mylar and Lee filter treatments). Each treatment was done in triplicate. For each replicate, 200 mL of culture was placed into a 1,200 mL UV transparent polyethylene Whirlpak bag. Bags where then exposed horizontally *in situ* to natural solar radiation near solar noon for 65 min at the highest intensity of the day (Hernández et al., 2016) and the irradiance of each spectral band was monitored (Supplementary Figure S1). The integrated irradiance corresponded to a biological dose of 2666.31 kJ m^-2^ for PAR, 144.38 kJ m^-2^ for UVA radiation, and 16.25 kJ m^-2^ for UVB radiation. After exposure bags were collected and stored in liquid nitrogen for transportation to laboratory facilities back in Antofagasta, Chile. Upon return to the lab, cultures from all the conditions were gradually thawed and centrifuged at 8000 g for 15 min at 4°C. Pellets were washed twice with 0.2 M sucrose to remove excess salt and then lyophilized until use.

### Laboratory Radiation Experiments

Experiments with artificial UVB radiation were performed using a UV chamber containing nine tubes of UVB lamps (UVB-313EL, Q-LAB Co, Westlake, OH, United States). The lamps have a maximum emission at 313 nm and we did not register a radiation emission below 280 nm (Supplementary Figure S2). The integrated irradiance of the lamps corresponded to a biological dose of 19.63 kJ m^-2^ (17.2% higher than the natural sunlight exposure in Salar de Huasco). For laboratory experiments pre-stationary phase cultures of *Rhodobacter* sp. were divided for two treatments; (i) UVB (ii) Dark (bags were wrapped with heavy black plastic). For each replicate 200 mL of culture was placed into a 1,200 mL Whirlpak bag and exposed horizontally *in vitro* for 65 min at 17 ± 1°C. Cultures were then centrifuged at 8000 g for 15 min at 4°C. Pellets were washed twice with 0.2 M sucrose to remove excess salt and freeze dried until use. Experiments were performed in quadruplicates for UVB treatment and in triplicates for dark controls.

### Viability Assay

Changes in viability during artificial UVB irradiation dose response curves for *Rhodobacter* sp. were determined using colony-forming units (CFU) counting and bacterial secondary production (BSP). CFU were determined for the artificial UVB radiation exposure experiment and its respective dark control by plating appropriate dilutions in triplicate on Marine agar. Counts were made after 5 days of incubation at 28°C and the percentage of CFU was determined as the ratio of CFU after treatment compared with initial cell counts. BSP of UVB irradiated samples were determinated by H^3^-Leucine incorporation following Smith and Azam (1992). Incubations were performed using 20 nM ^3^H-leucine for 1 hr post-exposure in the dark at room temperature. Logistics and field conditions prevented CFU and BSP determinations for the *in situ* solar exposures.

### Protein Extraction and Quantification

For protein extraction, the cell pellet was resuspended in one pellet volume of Lysis Buffer (6M guanidine chloride), and cells were mechanically lysed by sonication at 4°C (5 cycles of 10 s, amplitude 60%, 1 pulse rate). Lysed cells were centrifuged at 16,000 g at 4°C for 15 min and the supernatant fraction was used for analysis. Protein samples were reduced with 25 mM dithiothreitol (DTT) at 56°C for 30 min and alkylated with 50 mM iodoacetamide at room temperature for 30 min. Proteins were then precipitated with cold acetone overnight at -80°C, with an acetone/protein ratio of 4/1. The protein pellet was disolved in 100 mM phosphate buffer (pH 8) containing 2 M urea. Total protein concentration was determined by the Bradford method (Bradford, 1976) according to the manufacturer’s instructions, using bovine γ-globulin as a protein standard (Bio-Rad Protein Assay kit, Bio-Rad, Hertfordshire, United Kingdom). For LC-MS/MS analysis, a tryptic digestion (Sequencing grade modified trypsin, Promega) was performed overnight at 37°C, with an enzyme/substrate ratio of 1/25.

### Liquid Chromatography Tandem Mass Spectrometry (LC-MS/MS)

Protein identification and quantification were performed using a label-free strategy on an UHPLC-HRMS platform composed of an eksigent 2D liquid chromatograph and an AB SCIEX Triple TOF^TM^ 5600. Two separate LC-MS/MS runs were performed, one for Laboratory samples (7 samples) and one for Field samples (12 samples), thus protein identification and quantification were done separately for each experiment. Peptides were separated on a 25 cm C18 column (Acclaim pepmap 100, 3 μm, Dionex) by a linear acetonitrile (ACN) gradient [5–35% (v/v), in 15 or 120 min] in water containing 0.1% (v/v) formic acid at a flow rate of 300 nL min^-1^. Mass spectra (MS) were acquired across 400–1,500 m/z in high-resolution mode (resolution > 35000) with 500 ms accumulation time. The instrument was operated in DDA (Data Dependent Acquisition) mode and MS/MS were acquired across 100–1,800 m/z. A long run procedure was used to acquire quantitative data, and a duty cycle of 3 s per cycle was used to ensure that high quality extracted ion chromatograms (XIC) could be obtained. Protein searches were performed against *Rhodobacter* sp. genome (Pérez et al., unpublished), using ProteinPilot Software v4.1. Search parameters included differential amino acid mass shifts for oxidized methionine (+15.9949 Da). The identification of the overall set of proteins was validated by manual inspection of the MS/MS ion spectra, ensuring that a series of consecutive sequence-specific b- and y-type ions was observed. For quantification, the quant application of PeakView was used to calculate extracted ion chromatograms (XIC) for all peptides identified with a confidence > 0.99 using ProteinPilot^TM^. A retention time window of 2 min and a mass tolerance of 0.015 m/z were used. The area under the curve was exported in MarkerView^TM^, in which they were normalized based on the summed area of the entire run. MarkerView^TM^ enabled an average intensity for laboratory and field conditions to be calculated, as well as the significance of the difference between conditions based on a student *t*-test. Quantified proteins were kept with a *p*-value < 0.05 and with at least two peptides quantified with a *p*-value < 0.05. The false discovery rate (FDR) was calculated at the peptide level for all experimental runs using the Decoy option in Mascot; this rate was estimated to be lower than 1% using the identity threshold as the scoring threshold system. In order to be considered as well-quantified, proteins had to meet two criteria: fold change within the cut-off thresholds for a significantly differential regulation (1.5- or below 0.66-fold change in the artificial and natural light samples relative to the controls) and their *p*-value had to be < 0.05.

### Computational Analyses

A total of seven different conditions were generated by comparing quantified proteins of laboratory (artificial UVB treatment and dark control) and field experiments (Full Sun, PAR, PAR+UVA and dark treatment), allowing us to analyze the impact of each spectral band, namely: artificial UVB, natural Full Sun, natural PAR, natural PAR+UVA, natural UVA+UVB, natural UVA and natural UVB (**Table 1**). All proteins identified by proteomics were classified into 12 functional categories (Amino Acids Metabolism and Translation, Antioxidant, Cell Transport, Cell Division, Cell Envelope Biogenesis, Chaperones and Proteases, DNA Metabolism and Repair, Energy Production, Hypothetical Proteins, Sugar Transport, Transcription, Vitamins and Cofactors) according to functional descriptions from EggNOG 4.5 and Uniprot databases. Hierarchical cluster analysis (HCA) was developed using RStudio (ver. 0.99.879) software, for differentially regulated proteins from natural light treatments that were in common in at least two conditions.

**Table 1:** List of the analyzed conditions (artificial UVB, natural Full Sun, natural PAR, natural PAR+UVA, natural UVA+UVB, natural UVB, natural UVA), obtained by comparing different treatments and controls.

	Treatments	Replicates	Controls	Condition
ARTIFICIAL	UVB	4	DARK	UVB
	DARK	3	–	–
NATURAL	FULL SUN	3	DARK	FULL SUN
			PAR	UVA+UVB
			PAR+UVA	UVB
	PAR	3	DARK	PAR
	PAR+UVA	3	DARK	PAR+UVA
			PAR	UVA
	DARK	3	–	–

### Fluorescent Western Blot

Western blotting was used to confirm the changes in abundance of the well-conserved protein RecA detected by LC-MS/MS. Pelleted cells were lysed as described previously, and the supernatant was used for fluorescent western blotting analysis with ECL Plus (GE Healthcare). The protein samples were separated on a NuPAGE 4–12% Bis-Tris gel (Invitrogen), blotted onto a nitrocellulose membrane (Hybond-ECL; GE Healthcare), and probed with a mouse monoclonal antibody against RecA (1:1000 dilution; Clone ARM414, Clinisciences, France) as the primary antibody. A sheep secondary antibody anti-mouse HRP-conjugated F(AB)_2_ fragment (GE Healthcare) was added in a 1:5000 dilution. Membranes were scanned using a Typhoon FLA 9000 (GE Healthcare) and quantified using ImageJ version 1.50 analysis software (NIH).

## Results

**FIGURE 1:**
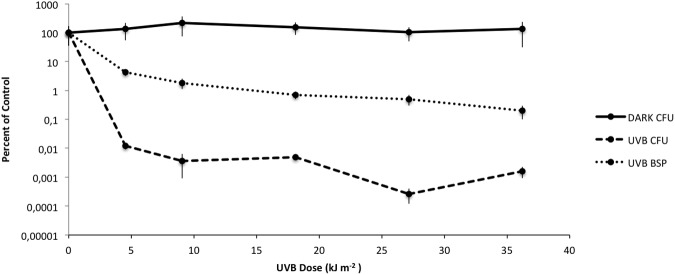
Changes in viability during artificial UVB irradiation in *Rhodobacter* sp., measured using CFU counting (DARK CFU, UVB CFU) and bacterial secondary production (UVB BSP). The data is expressed as the mean (± standard deviations) of three replicates compared to the unexposed control.

The viability of *Rhodobacter* sp. exposed to artificial UVB lamps, showed a gradual decrease in viability in the first 30 min of irradiation followed by a plateau of survival at 0.01% for UVB doses ranging from 9.81 to 19.63 kJ m^-2^. In contrast, cultures of *Rhodobacter* sp. maintained in the dark did not lose viability (**Figure 1**). Metabolic activity, as indicated by ^3^H-leucine incorporation, followed a similar pattern but was approximately two orders of magnitude higher when compared to unexposed controls (**Figure 1**).

### Differentially Regulated Proteins

**FIGURE 2:**
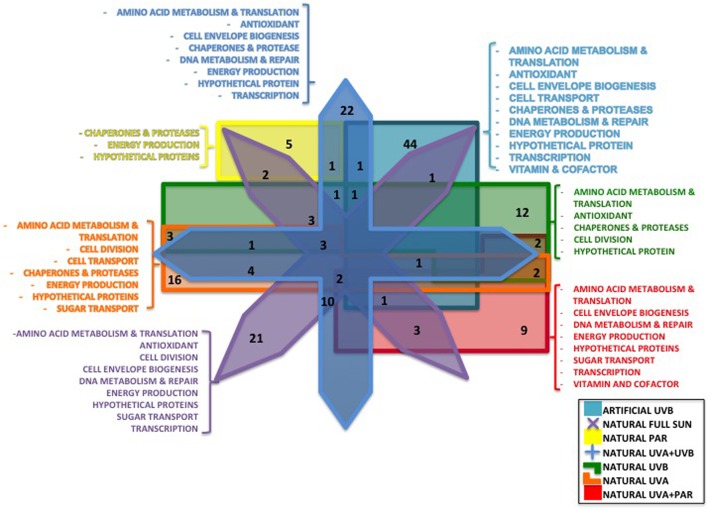
Venn diagram showing the number of differentially regulated proteins characterized in the different conditions: Artificial UVB (49 proteins), Natural Full Sun (48 proteins), Natural PAR (8 proteins), Natural PAR+UVA (19 proteins), Natural UVA+UVB (47 proteins), Natural UVB (27 proteins), and Natural UVA (29 proteins). Functional categories of the quantified proteins characterized in only one condition were listed.

A total of 957 proteins were identified (**Table 2** and Supplementary Data Sheets S1, S2) for artificial UVB treatment, and a total of 1195 proteins for natural light treatments (Supplementary Data Sheets S1, S2) to 17 and 20%, respectively, of the theoretical proteome of *Rhodobacter* sp. (4905 proteins). After combining both datasets, 1393 non-redundant proteins were characterized, representing 28.4% of the theoretical proteome of *Rhodobacter* sp. A total of 131 proteins were well quantified for the artificial UVB treatment and a total of 300 proteins for the natural light treatments (**Table 2**), leading to 313 non-redundant proteins after combining both datasets (Supplementary Tables S1, S2, S4). Of those well-quantified proteins, 171 showed a statistically significant differential regulation (Supplementary Table S3). After applying a commonly used cut-off thresholds of 0.66 and 1.5, a total of 49 proteins had statistically significant differential regulation compared to dark control for artificial UVB treatments (**Table 2**) and 178 proteins for natural light treatments (Full Sun, PAR, PAR+UVA, UVA+UVB, UVA and UVB) (**Figure 2** and **Table 2**). None of the quantified proteins were detected in all treatments and most of the differentially regulated proteins (127 proteins) were specific to one condition (**Figure 2**). In total, 42 proteins were found to be common in at least two conditions (**Figure 2**), among which only 10 proteins showed different regulation (see below in each corresponding section of the *Discussion*).

**Table 2:** Summary of the number of quantified proteins for the different conditions.

TREATMENT	Number of identified proteins	Number of quantified proteins	Number of quantified proteins within threshold		
			Total	Down-regulated (≤0.66)	Up-regulated (≥1.5)
Artificial UVB	957	131	49	18	31
Natural light treatments	1195	300	178	94	84
- Natural FULL SUN		72	48	29	19
- Natural PAR		21	8	2	6
- Natural PAR+UVA		36	19	13	6
- Natural UVA+UVB		70	47	29	18
- Natural UVB		46	27	13	14
- Natural UVA		55	29	8	21

**FIGURE 3:**
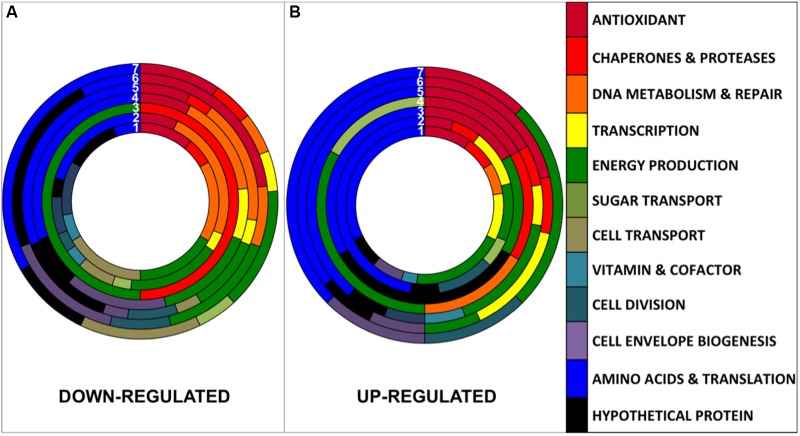
Comparison of the distribution of down-regulated **(A)** and up-regulated **(B)** proteins between the different conditions numbered from 1 to 7. (1) Artificial UVB (49 proteins), (2) Natural Full Sun (48 proteins), (3) Natural PAR (8 proteins), (4) Natural PAR+UVA (19 proteins), (5) Natural UVA+UVB (47 proteins), (6) Natural UVB (27 proteins), and (7) Natural UVA (29 proteins).

The 171 non-redundant proteins showing a statistically significant differential regulation (Supplementary Table S3) were classified into 12 functional categories (**Figure 3**). Artificial UVB treatment presented 49 differentially regulated proteins classified within 8 different functional categories (**Figure 3** and **Table 2**, circle N°1). In natural light treatments, Full Sun presented 48 proteins differentially expressed within 12 categories; UVA+UVB, 47 proteins within 11 categories; UVA, 29 proteins within 11 functional categories; UVB, 27 proteins within 11 categories; PAR+UVA, 19 proteins within 9 categories and PAR, 8 proteins within 5 categories (**Figure 3** and **Table 2**). Interestingly, UVB containing light treatments, namely artificial UVB, Full Sun and natural UVA+UVB presented the highest number of proteins differentially regulated (49, 48, and 47 proteins, respectively) and the broadest number of functional categories involved. Changes ranged from 0.39- to 2.79-fold for the artificial UVB treatment. In natural light treatments changes ranged from 0.16- to 5.44-fold for Full Sun, 0.07 to 15.65 for UVA+UVB, 0.13 to 23.7 for UVB, 0.04 to 2.71 for UVA treatments, 0.04 to 3.71 for PAR+UVA, and 0.27 to 1.83 for PAR treatment (Supplementary Table S3). The conditions of natural UVA+UVB and natural UVB showed the greatest changes among the up-regulated proteins, with an associated function of Antioxidant (ratio = 23.7 and ratio = 15.6, respectively; Supplementary Table S3). The following conditions: natural UVA+UVB, natural UVA and natural PAR+UVA, showed the greatest down-regulated proteomics ratios. Interestingly, both in natural UVA and natural PAR+UVA the protein of the Antioxidant functional category with the greatest down-regulated proteomic ratios was the glycine betaine/L-proline transport ATP-binding protein (RH1_01001, ratio = 0.04 and ratio = 0.04, respectively). In the condition natural UVA+UVB the protein of the functional category Energy Production, soluble hydrogenase 42 kDa subunit, presented the greatest down-regulated ratio (RH1_00494, ratio = 0.07).

### Comparison of Artificial and Natural UVB Conditions

**FIGURE 4:**
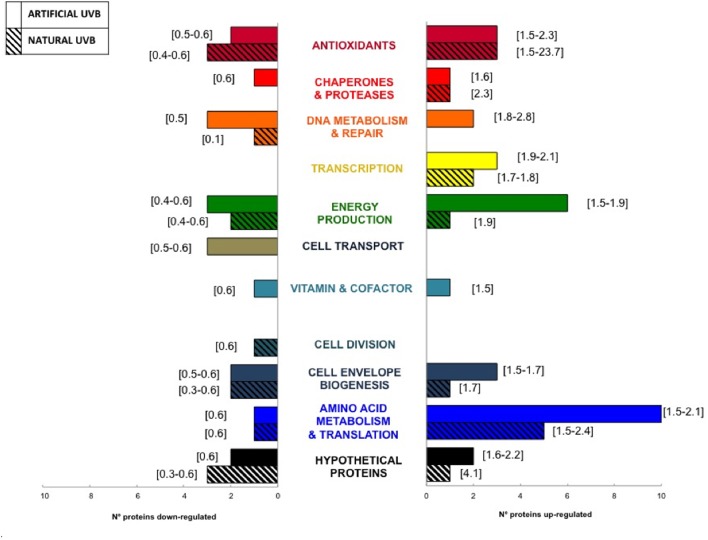
Comparison of the number of up-regulated (rightside histogram) and down-regulated (leftside histogram) proteins for both Natural and Artificial UVB conditions. Numbers into brackets represent the range of proteomics ratios for each functional category.

Our experimental design allowed us to compare the impact of natural and artificial UVB radiation on the protein distribution into functional categories (**Figure 4**). The comparison showed that most of the functional categories were represented in both conditions. Interestingly, the categories of Antioxidant, Amino Acids Metabolism and Translation, Energy Production and Transcription showed the greatest changes with regard to the up-regulated proteins for both artificial and natural UVB conditions, indicating an important role of those functions in the UVB response. Moreover, during UVB conditions, inositol monophosphatase (RH1_02008, ratio = 2.3 in artificial UVB, ratio = 6.1 natural UVB), involved in synthesis of the osmolyte inositol, the Energy Production related protein ATP synthase (RH1_01187, ratio = 1.78 in artificial UVB; RH1_01183, ratio = 1.86 in natural UVB) presented a significant up-regulation. On the other hand, cytochrome c subunits of energy related proteins (RH1_00669, ratio = 0.39 in artificial UVB and RH1_02355, ratio = 0.63 in natural UVB) and the hydrogen peroxide scavenger rubrerythrin (RH1_00544, ratio = 0.5 in artificial UVB) and peroxiredoxin (RH1_02575, ratio = 0.44 in natural UVB) presented a down-regulation. This set of proteins had the same regulation in both UVB conditions, showing a role as biomarkers for general UVB stress response. However, some differences were observed, such as a higher abundance in up-regulated proteins related with Energy Production (6 proteins) and Amino Acid Metabolism and Translation (10 proteins) categories in artificial UVB compared to natural UVB condition (1 and 5 proteins, respectively). Also, only three functional categories, comprising a very low number of proteins differentially regulated (1–3 proteins), were specific to one condition: Cell Transport (artificial UVB), Vitamin and Cofactor (artificial UVB) and Cell Division (natural UVB). Interestingly, within the Antioxidant category, a significant discrepancy in the ratios was observed between the natural and artificial UVB condition (1.5 to 2.3 and 1.5 to 23.7, respectively) in proteins such as the inositol monophosphatase and glycine betaine/L-proline transport.

### Comparison of Natural Light Conditions

A HCA was performed using changes of proteins found to be common in at least two natural light treatments (Full Sun, PAR, PAR+UVA, UVA+UVB, UVB and UVA). A total of 40 shared proteins within natural light treatments were used in the analysis, and were classified into 10 functional categories (Amino Acids Metabolism and Translation, Antioxidant, Cell Division, Cell Envelope Biogenesis, Cell Transport, Chaperone and Protease, DNA Metabolism and Repair, Energy, Transcription and Hypothetical Proteins).

**FIGURE 5:**
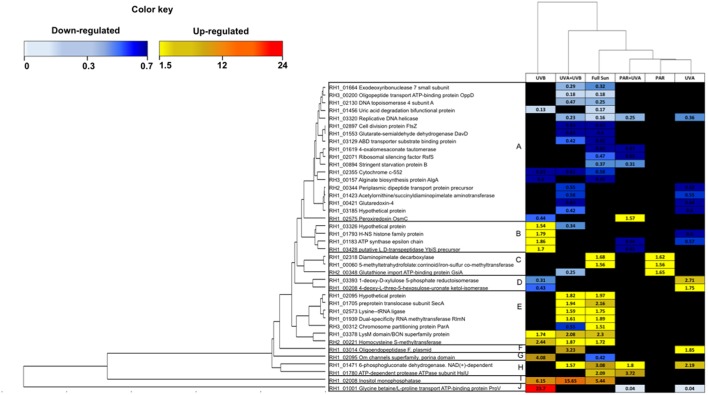
Heat-map showing 40 differentially regulated proteins shared between at least two treatments among the 6 Natural light conditions (Full Sun, PAR, PAR+UVA, UVA+UVB, UVA, UVB). Each cell of the grid was colored based on increased and decreased abundance above 1.5 or below 0.66, respectively.

Among the 40 common proteins, 10 distinct response patterns of protein abundance were revealed by HCA (**Figure 5**), which displayed a number of functional categories involved within each cluster. Cluster A was the largest (18 proteins), containing exclusively down-regulated proteins, involved in Amino Acid Metabolism and Translation, DNA Metabolism and Repair and Antioxidant during UVB, UVA, UVA+UVB and Full Sun light treatments. Cluster E, on the other hand, contained mainly up-regulated proteins involved in Amino Acid Metabolism and Translation, Antioxidant, Chaperone and Protease and Energy Production, during UVB, UVA+UVB and Full Sun conditions. The Clusters I and J, harboring only one protein in each cluster, presented the most important range of protein change ratios, with the proteins inositol monophosphatase (RH1_02008, ratio = 5.44 in Full Sun, ratio = 15.65 in UVA+UVB and 6.15 in UVB) and glycine betaine/L-proline transport ATP-binding protein ProV (RH1_01001, ratio = 23.7 in UVB, ratio = 0.04 in PAR+UVA and ratio = 0.04 in UVA), respectively.

This HCA revealed that the regulation under UVB light was more similar to the ones observed in UVA+UVB and Full Sun conditions than other spectral bands (**Figure 5**). UVA spectral band presented a distinct protein regulation pattern compared to UVB containing conditions, such as a significant down-regulation of Antioxidant protein glycine betaine transporter (RH1_01001, ratio = 0.04) and up-regulation of Energy Production proteins 1-deoxy-D-xylulose 5-phosphate reductoisomerase (RH1_03393, ratio = 2.71) and 4-deoxy-Lthreo-5-hexosulose-uronate ketol-isomerase (RH1_00208, ratio = 1.75), showing a qualitative similarity to PAR and PAR+UVA conditions (**Figure 5**). These conditions contained mostly proteins involved in Amino Acids Metabolism and Translation, Antioxidant and Energy Production categories (**Figure 5**).

### Quantification of RecA Protein Using Quantitative Western Blot

Surprisingly the RecA protein was found to be significantly down-regulated (0.52-fold change) in artificial UVB treatments compared to the dark control. A quantitative western blot confirmed this result with a significant down-regulation of RecA of 0.47-fold change in UVB treated cells compared to the dark control (*t*-test: *p*-value = 0.048; Supplementary Figure S3).

## Discussion

### Overview of Survival and Proteome Changes during Different Light Conditions

Artificial UVB exposure allowed us to characterize cell survival following UVB irradiation (**Figure 1**). BSP and survival curves are two different processes measured on two different time scales. BSP was determined within the 1st hour post-exposure and is an indication of relative cellular activity. In contrast, survival curves are based on a 72 h incubation that requires culturability, but does not measure other cellular activities. It is certainly likely that cellular activity can be measured in cells that are not ultimately culturable. The two different processes demonstrate very similar patterns, but on different scales, again demonstrating that short term activity is measurable and greater (when compared to control) than the ultimate measure of culturability/survival.

The results determinated an exponential decrease in cell viability at 4.9 kJ m^-2^ of exposure. Nevertheless, *Rhodobacter* sp. showed a plateau of survival at 0.01% for CFU and 1% BSP approximately for high UVB doses (9.81 to 19.63 kJ m^-2^). These results could represent an adaptation phase with an up-regulation of osmolytes, as a survival strategy, thus allowing the cells to better cope with the lethal effects of UVB radiation of the surviving population.

Cells exposed under light treatments containing UV radiation showed a greater change in protein regulation both in terms of protein number and relative abundance, in contrast to the PAR treatment, indicating that this condition had a lower impact on protein regulation compared to the UV-containing treatments (Supplementary Table S3). Interestingly, PAR+UVA, UVA, PAR treatments were grouped in the same cluster showing the highest similarity, indicating that the impact of UVA seemed more similar to PAR than UVB (**Figure 5**). The UVA spectral band presented a distinct protein regulation pattern compared to UVB conditions, displaying a qualitative similarity to the PAR condition (**Figure 5**). Indeed, shorter wavelengths, such as UVB radiation, are much more damaging to the cell compared to UVA and PAR spectral bands (Qiu et al., 2005; Rastogi et al., 2010; Matallana-Surget and Wattiez, 2013; Santos et al., 2013), which was confirmed in our proteomics study by quantitative (**Figure 3**) and qualitative analyses (**Figure 5**).

The comparison between artificial and natural UVB conditions (**Figure 4**) highlighted similarities in protein regulation of *Rhodobacter* sp. in both conditions, such as a significant up-regulation of proteins inositol monophosphatase (RH1_02008) and ATP synthase (RH1_01183; RH1_01187) and a down-regulation of cytochrome c subunits of energy production related proteins (RH1_00669; RH1_02355) and hydrogen peroxide scavengers (RH1_00544; RH1_02575). UVB stress response in this strain would comprise synthesis of a compatible solute such as inositol to cope with oxidative stress generated by exposure to harmfull UVB and ATP synthase would support its energetically costly synthesis. The biosynthesis of organic osmotic compounds to cope with osmotic stress, such as sucrose/trehalose or glycine-betaine described in several aerobic heterotrophic microorganisms is energetically costly (Oren, 1999), thus suggesting the need of increasing the ATP production to synthesize osmoprotectants, namely glycine betaine and inositol. Exposure to UVB-induced oxidative stress does not appear to lead to a significant increase in hydrogen peroxide in *Rhodobacter* sp., in view of the statistically significant down-regulation of the hydrogen peroxide scavengers rubrerythrin (RH1_00544, ratio = 0.5) and peroxiredoxin (RH1_02575, ratio = 0.44). Other antioxidants, however, were found to be key factors in *Rhodobacter* sp. to protect the cell from oxidative stress and those proteins are further discussed in the *Antioxidants* section. Interestingly, we observed that the response of *Rhodobacter* sp. exposed to natural UVB radiation was more diverse than the response under UVB lamps. The natural UVB treatment presented a significantly higher change range for osmoprotectants in the Antioxidant category compared to artificial UVB. This result indicates an underestimation of the UVB stress response observed using laboratory lamps when compared to *in situ* experiments in high UV ecosystems. Even though both treatments were not identical, in terms of UVB radiation doses (16.25 kJ m^-2^ and 19.63 kJ m^-2^ for natural UVB and artificial UVB, respectively), and with different control conditions (containing or not photoreactivating light: UVA/PAR, **Table 1**), our experimental design allowed us to characterize new biomarkers of resistance to UVB stress.

UVA+UVB and UVB conditions also presented a higher change ratio compared to the Full Sun treatment, which could be explained by the use of different control treatments to generate each condition. Indeed, while UVA+UVB and UVB conditions were obtained by comparing Full Sun treatment with PAR and PAR+UVA treatments, respectively, as controls, the impact of Full Sun was described by using a dark control (**Table 1**). *Rhodobacter* species are photoheterotrophic bacteria (Imam et al., 2013), known to use both light and organic carbon. Indeed, the genome of *Rhodobacter* sp. presented several genes involved in anoxygenic photosynthesis such as magnesium-protophyrin O-methyltransferase (*bchM*), light-independent protochlorophyllide reductase (*bchB, bchN*), and genes of puf operon (*pufQ, pufX*) (Pérez et al., unpublished). Thus, dark incubations without the addition of any carbon source could induce a starvation stress. In phototrophic bacteria, a loss of viability has been reported in starved cells during dark incubation compared to growth in presence of light (Kanno et al., 2014). Therefore, using a dark control could prove to be equally stressful for the cells and consequently mask some stress response proteins, thus underestimating the impact of UV-containing treatments.

### DNA Metabolism and Protection

**FIGURE 6:**
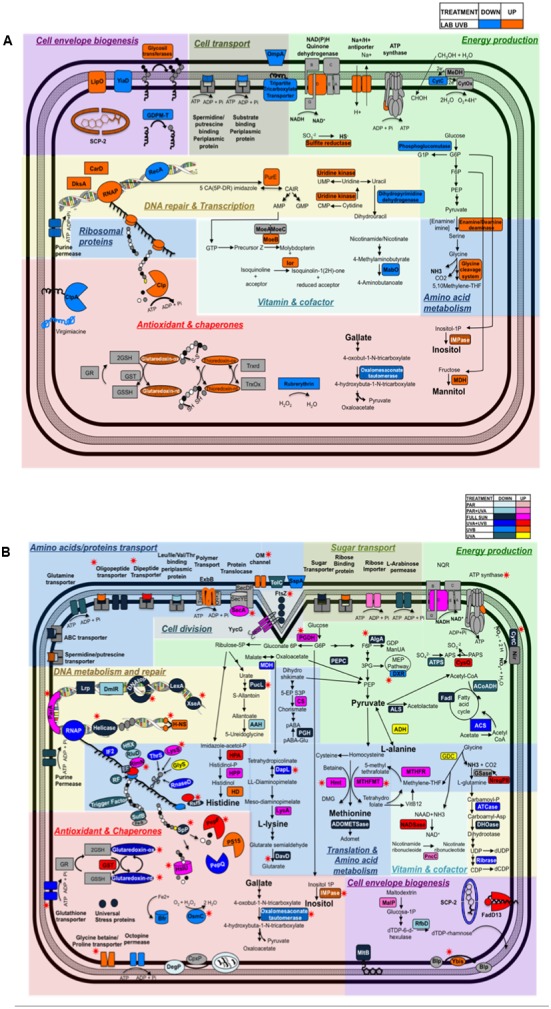
Diagram depicting the cellular pathways impacted by **(A)** Artificial UVB treatment and **(B)** Natural light conditions (Full Sun, PAR, PAR+UVA, UVA+UVB, UVB, UVA) in *Rhodobacter* sp. The up and down-regulated proteins are represented according to the color code provided at the top right corner of the diagram. Striped boxes represent proteins shared by Artificial UVB treatment and Natural light treatments. Red stars indicate proteins shared by more than one condition within among all natural light treatments.

One of the most surprising findings of our study was the significant down-regulation of the RecA protein (RH1_00148, ratio = 0.52) under artificial UVB radiation compared to dark control (**Figure 6A**, Supplementary Figure S3, and Table S3). However, bacteria exposed to natural high UV radiation did not show any differential regulation of the RecA protein, and instead protein LexA (RH1_00819, ratio = 0.55) was found to be down-regulated (**Figures 6A,B** and Supplementary Table S3). It is well known that solar radiation generally induces an overexpression of the RecA protein, both in bacterial isolates and natural communities (Booth et al., 2001a,b; Matallana-Surget et al., 2012a). Nevertheless, in accordance with our findings, Kolowrat et al. (2010) found a down-regulation of both *recA* and *lexA* transcripts in the marine cyanobacterium *Prochlorococcus marinus* during UV exposure, with a delay in DNA synthesis and replication. Although RecA is the sole protein required for LexA auto-cleavage in *Deinococcus radiodurans*, it has been shown that LexA does not regulate the radiation-dependent induction of RecA in *D. radiodurans* (Narumi et al., 2001), and this could also be the case in *Rhodobacter* sp. exposed to artificial UVB radiation. Nevertheless, more experiments targeting the expression of RecA, LexA and SOS genes would be needed to better understand the SOS response in *Rhodobacter*.

Several DNA binding proteins were found to be differentially regulated after artificial and natural light treatments, which could play an important role in chromosome condensation and DNA protection in a RecA-independent response. The histone-like H-NS protein (RH1_01793, ratio = 1.79) and two transcriptional regulators, DksA (RH1_00464, ratio = 2.08) and CarD (RH1_03027, ratio = 2.04), were found to be up-regulated in artificial UVB treatments (**Figure 6A**). H-NS, involved in the condensation of bacterial chromosome, could participate in the RecA-independent response mechanism in *Rhodobacter* sp., by bringing in close proximity two homologous region in a DNA molecule (Dame et al., 2000; Joyeux and Vreede, 2013). H-NS could also modify the nucleoid structure, acting as a physical shield protecting against DNA damage (Anuchin et al., 2010). H-NS protein was also found to be down-regulated (ratio = 0.6) in the UVA condition, which could indicate higher level of DNA damage induced by UVB compared to UVA radiation (Ravanat et al., 2001; Rastogi et al., 2010) thus demonstrating a high specificity in the response of *Rhodobacter* sp. to each individual spectral band. DksA and CarD allow bacteria to cope with DNA damage and oxidative stress in stress conditions such as UV radiation exposure by protecting the genome against instability (Mogull et al., 2001; Trautinger et al., 2005; Furman et al., 2012; Zenkin and Yuzenkova, 2015). Indeed, Tehranchi et al. (2010) demonstrated that DksA inhibited transcriptional pausing by interfering with DNA replication, preventing the recruitment of RecA and thus the activation of the SOS response. Similarly, the recently identified CarD factor in *Mycobacterium tuberculosis* is DNA damage-inducible (Stallings et al., 2009), and would have the same role as DksA (Srivastava et al., 2013; Bae et al., 2015). Moreover, the proteins exoribonuclease VII, involved in mismatch repair (MMR) (Repar et al., 2013), (RH1_01664, ratio = 0.32), replicative DNA Helicase (RH1_03320 ratio = 0.16 for UVA and ratio = 0.23 for UVA+UVB), DNA topoisomerase IV (RH1_02130, ratio = 0.25 for Full Sun and ratio = 0,47 for UVA+UVB), and the two transcriptional regulators Lrp (RH1_01430, ratio = 0.58) and DmIR were found to be down-regulated (RH1_00417, ratio = 0.62) after natural light treatments (**Figure 6B** and Supplementary Table S3). Down-regulation of this protein could indicate a preference for bacterial chromosome condensation, by repressing enzymes that could modify the nucleoids conformation, such as DNA helicase and DNA topoisomerase proteins (Duguet, 1997). Interestingly, it was also previously shown that *recA* gene repression regulate the *umuDC*-dependent cell cycle checkpoint, giving cells more time to repair DNA damage (Opperman et al., 1999; Kolowrat et al., 2010).

We also observed in artificial UVB exposed cells an increase in two enzymes involved in nucleotide biosynthesis, uridine kinase (RH1_01523, ratio = 1.81) (Tomoike et al., 2015) and the enzyme 5-(carboxyamino) imidazole ribonucleotide mutase (PurE, RH1_01437, ratio = 2.79; Mueller et al., 1994). Odsbu et al. (2009) observed in *E. coli* that replication forks can move slower than normal or even be stalled if there was a severe limitation in dNTPs, which could lead to genome instability, specially under damaging UV exposure where the cells can have an increase on nucleotide demands to repair DNA strand breaks. These enzymes could avoid fork stalling that could threat the genome integrity and could thus reduce the risk of UV-induced replication error.

### Key Role of Antioxidants to Cope with Oxidative Stress

The most striking result of our study was the importance of osmoprotectants, commonly involved in osmotic stress response to actively cope with damaging UV radiation. Although all UV exposure of *Rhodobacter* sp. were performed using a standard microbiological medium with low salt concentration (0.5% NaCl) for both field and laboratory experiments, it is important to remember that our bacterial model was isolated from a saline Andean wetland, a hostile habitat with great desiccation levels and high salt concentration. In this way, *Rhodobacter* sp. might have evolved an efficient strategy involving osmoprotectants and compatible solutes to adapt to large fluctuations in osmolarity, as reported in *Tistlia consotensis*, a halotolerant bacterium isolated from an Andean saline environment (Rubiano-Labrador et al., 2014) and in the photoheterotrophic bacterium, *Rhodobacter sphaeroides* (Tsuzuki et al., 2011). In our study, we suggest that osmoprotectants, mainly glycine betaine and inositol could act as general stress protectants to allow the resistance of *Rhodobacter* sp. to high UV radiation doses. In that respect, several studies indicated that these molecules have multiple properties, confering protection againgst oxidative, osmotic and temperature stresses by stabilizing the proteins and lipids (Boscari et al., 2002; Castenholz, 2004; Ly et al., 2004; Glaeser and Klug, 2005; Gao and Garcia-Pichel, 2011; Hoffmann and Bremer, 2011; Pilonieta et al., 2012; Mykytczuk et al., 2013). The extremophile *Halobacterium salinarum* was found to be resistant to ionizing radiation as a fortuitous consequence of a high tolerance to osmotic stress (Fredrickson et al., 2008; Kish et al., 2009). Moreover, studies have suggested that the strong robustness of *D. radiodurans* to ionizing radiation was an incidental consequence of its adaptation to desication and strong oxidative stress resistance (Mattimore and Battista, 1996). Thus, our results agree with previous studies, showing the molecular adaptation of an extremophile microorganism under multiple stresses encountered in a hostile habitat such as Salar de Huasco.

A significant number of enzymes related to the synthesis and transport of compatible solutes were found to be up-regulated in cells exposed to both artificial and natural light treatments, exhibiting the largest proteomics ratios among all the differentially regulated proteins. Indeed, proteins involved in the transport of osmoprotectants such as glycine betaine/L-proline transport ProV (RH1_01001) showed a surprisingly high 23.7-fold change in protein abundance in natural UVB treatment (**Figure 6B**). Nevertheless, these proteins also presented a down-regulation in PAR+UVA (ratio = 0.04) and UVA (ratio = 0.04) conditions, compared to both dark control and PAR treatment, respectively. Because extremophilic microorganisms have specialized adaptations that allow them to live in extreme conditions (e.g., salt, temperature, pH, pressure), many extremophiles cannot survive in moderate environments (e.g., Cayol et al., 2000; Thombre et al., 2016). In that respect, growth in the total absence of UV radiation might be experienced as a stress in *Rhodobacter* sp. Furthermore, it has been reported that dark conditions in starved cells can increase the stress response of photoheterotrophic bacteria adapted to high light intensities (Kanno et al., 2014). This could also explain why in the response to Full Sun and artificial UVB treatments (both generated using dark controls) we did not observe the differential expression of the glycine betaine proteins in comparison to the natural UVB treatment.

Inositol monophosphatase (RH1_02008) involved in the biosynthesis of inositol (Matsuhisa et al., 1994), was also found to be strongly up-regulated, showing 2.3-fold change in artificial UVB, 5.4 in Full Sun, 6.1 in UVB and 15.6 in UVA+UVB conditions. Previous studies have reported that inositol containing molecules could act as a glutathione molecule in mycobacteria (Majumder and Biswas, 2006) and as an osmolyte in hyperthermophile archaea (Chen et al., 1998). It is worth mentioning that in all of these treatments, UVB radiation was part of the spectral band, which certainly can be related to an increased oxidative stress under UV containing treatment compare to the impact of PAR and dark conditions.

A number of other proteins involved in the production and transport of compatible solutes were found to be significantly up-regulated, such as a carnitine transport system (RH1_00640, ratio = 2.45) under natural UVA+UVB radiation and mannitol deshydrogenase (RH1_00206, ratio = 1.7) involved in biosynthesis of mannitol in artificial UVB treatment. Carnitine and mannitol can act as compatible organic osmolytes and free radical scavengers (Shen et al., 1997; Efiuvwevwere et al., 1999; Peluso et al., 2000; Wisselink et al., 2002; Conde et al., 2011; Rinaudo et al., 2014).

Finally, both glutaredoxin (RH1_00421) and thioredoxin (RH1_01783) were found to be up-regulated (ratio = 1.52, ratio = 1.64, respectively) in artificial UVB treatment. These enzymes are responsible for maintaining a cellular reducing environment, catalyzing thiol-disulfide exchange reactions (Zeller and Klug, 2006), and also are involved in DNA synthesis and protein repair (Berndt et al., 2008; Zahedi Avval and Holmgren, 2009; Sengupta and Holmgren, 2014). Even though under natural UVA+UVB conditions glutaredoxin was found to be down-regulated (ratio = 0.63), the protein glutathione transferase (RH1_00964, ratio = 1.6) was up-regulated, indicating a participation of the glutathione system in the response to UV radiation in *Rhodobacter* sp. Furthermore, a superoxide dismutase precursor (RH1_ 00450) was found to be up-regulated under natural PAR condition (ratio = 1.55). Similar up-regulation of an Iron-SOD were found in *Sphingopyxis alaskensis* under PAR and PAR+UVA treatments, suggesting that this enzyme may facilitate defende against free radical and catalyze the dismutation of toxic superoxide into oxygen and hydrogen peroxide (Matallana-Surget et al., 2009). Moreover, Kurth et al. (2015) reported an up-regulation of catalase on *Acinetobacter* sp. V3, isolated from an Argentinian altiplanic environment, demonstrating the importance of antioxidants as a first defense line in the response of UVB stress.

### Protein Metabolism: Synthesis and Turnover

In contrast to chaperones that prevent protein unfolding or rescue unfolded proteins, proteolytic activity and proteases are important for processing irreversibly damaged or denatured proteins (Rawlings and Barrett, 1994; Kress et al., 2009). The proteases-chaperones Clp (RH1_03216, ratio = 1.55) and HslU (RH1_01780, ratio = 2.09) showed an increased in abundance in artificial UVB and Full Sun treatments, respectively (**Figures 6A,B**). Two peptidases exhibited an increase in abundance following natural UV treatments as follows: oligopeptidase F (RH1_03014, ratio = 3.23) was up-regulated in the UVA+UVB treatment and peptidase S15 (RH1_01065, ratio = 2.03) up-regulated in the UVB treatment.

The expression of proteins involved in amino acids metabolism was up-regulated for both artificial and natural light treatments (**Figure 6** and Supplementary Table S3). An increased abundance of amino acids would allow a faster replacement of damaged and degraded proteins as a result of damaging UV radiation. Also in both conditions (artificial and natural UV) glycine cleavage system (glycine cleavage system H protein, RH1_02462, ratio = 1.69 and glycine dehydrogenase, RH1_02464, ratio = 1.49, respectively) were up-regulated (**Figure 6A**). The glycine cleavage system aims at converting glycine into ammonium and methylene tetrahydrofolate (THF), a coenzyme used in thymidylate synthesis to produce thymidine and also involved in methionine biosynthesis (Kikuchi et al., 2008). Interestingly, amino acids derivatives might play another key role in the resistance of *Rhodobacter* sp. by acting as osmoprotectants (Sleator and Hill, 2002) and thus protecting the cell against oxidative stress.

### Energy Production

We observed the repression of energy pathways such as the fatty acid cycle (acetyl CoA acetyltransferase, RH1_02467, ratio = 0.43, Full Sun and acyl-CoA dehydrogenase, RH1_01807, ratio = 0.56, UVA; **Figures 6A,B** and Supplementary Table S3) and methylotrophic metabolism (methanol dehydrogenase [cytochrome c] subunit 1 precursor, RH1_00669, ratio = 0.39, artificial UVB) under UV-containing sunlight treatments, indicating a preference to a redirection of the majority of carbon sources available in the cell to enter anabolic pathways linked to the synthesis of bio-molecule backbones such as amino acids and nucleotides, and most notable osmoprotectants such as inositol, gallate and mannitol (**Figures 6A,B**).

Methylotrophic bacteria are able to utilize reduced carbon compounds containing one or more carbon atoms that contain no carbon-carbon bonds (such as methane, methanol, and other methylated compounds) as their carbon and energy source (Chistoserdova et al., 2009). On the other hand, UVB-containing treatments (artificial UVB, natural Full Sun and UVB treatments), led to an increase in abundance of proteins involved in other general energy production pathways such as NADPH dehydrogenase (quinone; RH1_01139, ratio = 1.5, artificial UVB), ATP synthase subunit delta (RH1_01187, ratio = 1.78, artificial UVB), NADH-quinone oxidoreductase subunit D (RH1_00737, ratio = 1.81, natural Full Sun), and ATP synthase epsilon chain (RH1_01183, ratio = 1.86, natural UVB) (**Figures 6A,B**).

Photoheterotrophic bacteria can effectively survive in natural enviroments in which light energy is available even if organic sources are depleted due to a highly versatile metabolism, regulating ATP production and utilization to maintain cell viability (Zong and Jiao, 2012). It has been reported that purple non-sulfur bacteria under starvation stress support a longer survival by maintaining ATP levels in the cultures using photosynthesis, compared to cultures with a decreased viability in dark conditions (Kanno et al., 2014). Although we identified proteins involved in photosystem (PS) regulation such as the photosynthetic apparatus regulatory protein RegA (RH1_01792) in artificial and natural light treatments, we did not quantify proteins related to photosynthetic complexes in our study. This could be explained by the fact that at high oxygen concentration (∼21%), this photoheterotrophic bacterium would lack photosynthetic complexes and uses aerobic respiration for energy generation (Zeller et al., 2005) due to a high sensitivity of PS to oxidative damage (Nishiyama et al., 2005). Light-regulated metabolic versatility in this photoheterotrophic strain could also be observed in a down-regulation of the enzyme acetyl-coA synthetase (RH2_00279, ratio = 0.66, UVA+UVB condition), involved in acetate assimilation, indicating that during the PAR light condition (used as control), this alternative pathway would have an important role in anabolic processes (Imam et al., 2013; Leroy et al., 2015).

Stress responses involving biosynthesis of osmoprotectants, amino acids, ATP-dependent proteases/chaperones are ATP-coupled energetically costly reactions, and require a high energetic demand to cope with the UV stress. *Rhodobacter* sp. would prefer using energy production pathways involved in electron transport that could fullfill high energy requirements after UVB treatments in a faster and more efficient manner. Indeed, electron transport is the most productive process compared to the rest of pathways in cellular respiration (34 ATP molecules).

### Extreme Habitat and Climate Change: Impact of Increase Ultraviolet Radiation

The discovery of the ‘ozone hole’ over Antarctica in the 1980’s heralded research into the harmful effects of increasing levels of solar UV radiation, particularly UVB (280–320 nm) that damages many different cellular components (Farman et al., 1985). In 2011, the alarm sounded again on a thinning ozone layer as the Arctic region attained record low ozone levels. By the end of the 21st century, UVB radiation at the surface of the earth will have increased by approximately 5–10% in temperate latitudes and by 20% for high latitudes (Zepp et al., 2011), thus potentially affecting a significant proportion of organisms. Bacteria and phytoplankton are the basis of the food web and biochemical cycles, and any changes in their current community structure and function are of great ecological importance. Understanding microbial responses to environmental change is urgently needed. Because of their large population sizes and short generation times, microbial populations can respond rapidly to environmental change. Radiation affects phytoplankton and heterotrophic bacteria by altering the predominance of many species, their diversity, and potentially their role in ecosystem function, therefore having critical implications for changes to global primary production.

In the context of future climate change, the study of extreme environments such as Salar de Huasco helps to better anticipate the impact of a predicted increase in UVB radiation reaching the Earth’s surface. Very importantly, we demonstrated that natural UVB treatments presented higher changes of protein expression both qualitatively and quantitatively compared to artificial UVB lamps, confirming the need to perform more *in situ* experiments in extreme environments. The characterisation of protein regulation of microbial communities using a metaproteomics approach seems to be an appropriate technology to more accurately reflect the major functions occurring in this extreme environment. Future experiments will be performed to determine the combined effect of high solar radiation and salt stress on natural microbial communities isolated from Salar de Huasco.

## Conclusion

Our knowledge of photoheterotrophic bacteria is still very limited especially for high altitude ecosystems, where photoheterotrophy may have evolved very specific mechanisms to cope with extreme doses of damaging solar radiation. Under these conditions, the identification of molecular mechanisms of UV radiation stress response in microbial communities will contribute to understanding the strategies used to repair and protect against UV-induced damage.

Our data demonstrated the ability of *Rhodobacter* sp. to use different types of energy production to fulfill the energy demands for the synthesis of compatible solutes to prevent UV induced oxidative stress. This indicates a strong adaptation of *Rhodobacter* sp. to detrimental and changing conditions in poly-extreme environments. We cannot rule out the mutagenicity of DNA damage repair processes in microorganisms and microbial communities, which could explain the observed down regulation of RecA and may suggest the prevalence of other response pathways, such as physical DNA protection and antioxidant osmoprotectants.

The efficiency in the production of enzymes involved in the synthesis of osmolytes could be applied for biotechnological purposes using *Rhodobacter* sp. as a source for production of natural commercial osmoprotectant compounds. Finally, the response of *Rhodobacter* sp. analyzed in this study in liquid cultures might be different in a solid microbial mat assembly and under anaerobic conditions, suggesting future studies at the molecular and ecological levels.

## Author Contributions

VP executed field and laboratory experiment, analyzed proteomic and viability data, and wrote the paper. SM-S supervised proteomic experiments, analyzed data and wrote the paper. MH contributed in paper writing, designed experimental setup and participated in field experiment. LK executed field and laboratory experiments. CD and VM contributed in paper writing and are PIs of project grants supporting fieldtrip and data analyses. RW executed LC-MS/MS runs. WJ contributed in paper writing, data analysis, executed solar and artificial UVB radiation measurements and bacterial secondary production determination.

## Conflict of Interest Statement

The authors declare that the research was conducted in the absence of any commercial or financial relationships that could be construed as a potential conflict of interest.
