# Supplementary material for: Bacterial Survival under Extreme UV Radiation: A Comparative Proteomics Study of Rhodobacter sp., Isolated from High Altitude Wetlands in Chile
Source: Front Microbiol. 2017 Jun 26;8:1173. doi: 10.3389/fmicb.2017.01173 (PMC5483449; doi:10.3389/fmicb.2017.01173)
Supplement: Supplementary file 3 [file Table_3.DOCX]

**Table S3.** Fold change ratios of the 171 non-redundant differentially regulated proteins.Artificial UVB (49 proteins), Natural FULL SUN (48 proteins), Natural PAR (8 proteins), Natural PAR+UVA (19 proteins), Natural UVA+UVB (47 proteins), Natural UVB (27 proteins) and Natural UVA (29 proteins). P-value are shown in brackets. Each cell of the grid was coloured based on increased abundance above 1.5 or below 0.66 relative to their respective controls, and the level of reproducibility of the data; more reproducible (p-value<0.01) or less reproducible (0.01<p-value<0.05). Proteins with asterisk were re-annotated, for more information see Table S4.

| Abundance ratio >1.5 (p-value <0.01) |  |
| --- | --- |
| Abundance ratio >1.5 (p-value >0.01) |  |
| Abundance ratio < 0.66 (p-value <0.01) |  |
| Abundance ratio < 0.66 (p-value >0.01) |  |

|  | **PROTEINS DIFFERENTIALLY EXPRESSED** | **Art UVB** | **Nat FS** | **Nat PAR** | **Nat PAR+UVA** | **Nat UVB+UVA** | **Nat UVB** | **Nat UVA** |
| --- | --- | --- | --- | --- | --- | --- | --- | --- |
| 1 | RH1_00017 GTPase HflX |  |  |  |  |  |  | **0.57 (0.00845)** |
| 2 | RH1_00035 30S ribosomal protein S2 |  |  |  |  | **0.61 (0.03247)** |  |  |
| 3 | RH1_00060 5-methyltetrahydrofolate:corrinoid/iron-sulfur protein co-methyltransferase |  | **1.56 (0.00659)** | **1.56 (0.00709)** |  |  |  |  |
| 4 | RH1_00077 FeS cluster assembly protein SufB |  |  |  |  |  |  | **0.62 (0.01283)** |
| 5 | RH1_00146 hypothetical protein |  |  |  |  | **0.34 (0.01619)** | **0.35 (0.02559)** |  |
| 6 | RH1_00148 recombinase A | **0.52 (0.01539)** |  |  |  |  |  |  |
| 7 | RH1_00168 4-methylaminobutanoate oxidase | **0.63 (0.00151)** |  |  |  |  |  |  |
| 8 | RH1_00180 putative periplasmic serine endoprotease DegP |  |  | **0.27 (0.04742)** |  |  |  |  |
| 9 | RH1_00206 Polyol:NADP oxidoreductase | **1.71 (0.02567)** |  |  |  |  |  |  |
| 10 | RH1_00208 4-deoxy-L-threo-5-hexosulose-uronate ketol-isomerase |  |  |  |  |  | **0.43 (0.04201)** | **1.75 (0.041)** |
| 11 | RH1_00339 L-arabinose transport system permease protein |  |  |  |  |  |  | **0.29 (0.01964)** |
| 12 | RH1_00345 Carbon monoxide dehydrogenase large chain |  | **0.65 (0.02445)** |  |  |  |  |  |
| 13 | RH1_00351 Phosphoglucomutase | **0.64 (0.00441)** |  |  |  |  |  |  |
| 14 | RH1_00356 Maltodextrin phosphorylase |  |  |  | **1.56 (0.02257)** |  |  |  |
| 15 | RH1_00398 50S ribosomal protein L32 | **1.72 (0.01962)** |  |  |  |  |  |  |
| 16 | RH1_00417 HTH-type transcriptional regulator DmlR |  |  |  | **0.62 (0.03999)** |  |  |  |
| 17 | RH1_00421 Glutaredoxin-4 | **1.52 (0.00351)** |  |  |  | **0.63 (0.01744)** |  | **0.64 (0.00597)** |
| 18 | RH1_00464 RNA polymerase-binding transcription factor DskA | **2.08 (0.01215)** |  |  |  |  |  |  |
| 19 | RH1_00494 Soluble hydrogenase 42 kDa subunit |  |  |  |  | **0.07 (0.0068)** |  |  |
| 20 | RH1_00497 Elongation factor P |  | **0.61 (0.01665)** |  |  |  |  |  |
| 21 | RH1_00544 Rubrerythrin | **0,50 (0.03059)** |  |  |  |  |  |  |
| 22 | RH1_00571 Putative Sulfite reductase |  |  |  |  | **0.55 (0.00361)** |  |  |
| 23 | RH1_00640 Carnitine transport ATP-binding protein OpuCA |  |  |  |  | **2.45 (0.02404)** |  |  |
| 24 | RH1_00660 Putative Cell division MukB domain |  |  |  |  |  |  | **1.78 (0.04919)** |
| 25 | RH1_00665 Virginiamycin B lyase | **0.64 (0.0000759)** |  |  |  |  |  |  |
| 26 | RH1_00669 Methanol dehydrogenase [cytochrome c] subunit | **0.39 (0.0043)** |  |  |  |  |  |  |
| 27 | RH1_00716 Peptide chain release factor 3 |  |  |  |  |  |  | **0.41 (0.05923)** |
| 28 | RH1_00731 Putative lipoprotein | **1.70 (0.03454)** |  |  |  |  |  |  |
| 29 | RH1_00737 NADH-quinone oxidoreductase subunit D |  | **1.81 (0.03929)** |  |  |  |  |  |
| 30 | RH1_00759 outer membrane channel protein |  |  |  |  |  |  | **0.63 (0.00957)** |
| 31 | RH1_00819 LexA repressor |  | **0.55 (0.02501)** |  |  |  |  |  |
| 32 | RH1_00845 Xaa-Pro dipeptidase |  |  |  |  | **0.61 (0.03172)** |  |  |
| 33 | RH1_00849 Ribonuclease D |  |  |  |  | **0.54 (0.03222)** |  |  |
| 34 | RH1_00888 Spermidine/putrescine-binding periplasmic protein precursor |  |  |  |  |  | **1.65 (0.03344)** |  |
| 35 | RH1_00894 Stringent starvation protein B |  | **0.37 (0.00134)** |  | **0.31 (0.02834)** |  |  |  |
| 36 | RH1_00945 Hypothetical protein |  | **1.97 (0.03307)** |  |  | **1.82 (0.05499)** |  |  |
| 37 | RH1_00947 Putative Outer membrane autotransporter barrel family |  |  |  |  |  | **1.54 (0.04892)** |  |
| 38 | RH1_00964 Glutathione S-transferase GST-4.5 |  |  |  |  | **1.6 (0.00321)** |  |  |
| 39 | RH1_01001 Glycine betaine/L-proline transport ATP-bindi |  |  |  | **0.04 (0.00251)** |  | **23.7 (0.03041)** | **0.04 (0.00369)** |
| 40 | RH1_01009 5,10-methylenetetrahydrofolate reductase |  | **2.46 (0.0077)** |  |  |  |  |  |
| 41 | RH1_01044 Histidinol-phosphatase |  | **1.80 (0.02485)** |  |  |  |  |  |
| 42 | RH1_01065 Peptidase S15 |  |  |  |  |  | **2.03 (0.01801)** |  |
| 43 | RH1_01105 Na(+)/H(+) antiporter subunit A | **1.66 (0.02378)** |  |  |  |  |  |  |
| 44 | RH1_01139 NAD(P)H dehydrogenase (quinone) | **1.50 (0.00679)** |  |  |  |  |  |  |
| 45 | RH1_01141 Glutamine-dependent NAD(+) synthetase |  |  |  |  | **1.7 (0.05436)** |  |  |
| 46 | RH1_01147 Rod shape-determining protein MreB |  |  |  |  |  | **0.62 (0.05653)** |  |
| 47 | RH1_01183 ATP synthase epsilon chain |  |  |  | **0.64 (0.04337)** |  | **1.86 (0.01568)** | **0.57 (0.00598)** |
| 48 | RH1_01187 ATP synthase subunit delta | **1.78 (0.03352)** |  |  |  |  |  |  |
| 49 | RH1_01197 Histidinol-phosphate aminotransferase 2 |  |  |  |  | **1.63 (0.03338)** |  |  |
| 50 | RH1_01294 META domain protein |  |  |  | **0.47 (0.00301)** |  |  |  |
| 51 | RH1_01381 putative hydrolase |  |  |  | **0.66 (0.00441)** |  |  |  |
| 52 | RH1_01415 50S ribosomal protein L28 | **1.77 (0.01137)** |  |  |  |  |  |  |
| 53 | RH1_01423 Acetylornithine/succinyldiaminopimelate amino |  |  |  |  | **0.58 (0.04431)** |  | **0.55 (0.00628)** |
| 54 | RH1_01430 Leucine-responsive regulatory protein |  | **0.58 (0.01757)** |  |  |  |  |  |
| 55 | RH1_01437 N5-carboxyaminoimidazole ribonucleotide mutas | **2.79 (0.0035)** |  |  |  |  |  |  |
| 56 | RH1_01456 Uric acid degradation bifunctional protein |  | **0.17 (0.00416)** |  |  |  | **0.13 (0.00724)** |  |
| 57 | RH1_01466 Bacterioferritin |  |  |  |  |  | **0.57 (0.04965)** |  |
| 58 | RH1_01471 6-phosphogluconate dehydrogenase, NAD(+)-dependent, decarboxylating |  | **3.08 (0.02361)** |  | **1.80 (0.02513)** | **1.57 (0.01554)** |  | **2.19 (0.00281)** |
| 59 | RH1_01499 Malate dehydrogenase |  |  |  |  | **0.64 (0.0403)** |  |  |
| 60 | RH1_01523 Uridine kinase | **1.81 (0.00759)** |  |  |  |  |  |  |
| 61 | RH1_01540 Aspartate carbamoyltransferase |  |  |  |  | **0.5 (0.03908)** |  |  |
| 62 | RH1_01553 Glutarate-semialdehyde dehydrogenase DavD |  | **0.60 (0.00461)** |  |  | **0.63 (0.02418)** |  |  |
| 63 | RH1_01563 ATPase family associated with various cellular activities (AAA) |  |  |  |  | **2.54 (0.04948)** |  |  |
| 64 | RH1_01574 putative lipoprotein YiaD precursor | **0.62 (0.03428)** |  |  |  |  |  |  |
| 65 | RH1_01619 4-oxalomesaconate tautomerase | **0.52 (0.03475)** | **0.64 (0.03306)** |  | **0.67 (0.03687)** |  |  |  |
| 66 | RH1_01621 Tripartite tricarboxylate transporter family | **0.62 (0.00419)** |  |  |  |  |  |  |
| 67 | RH1_01657 Chorismate synthase |  | **2.79 (0.03329)** |  |  |  |  |  |
| 68 | RH1_01664 Exodeoxyribonuclease 7 small subunit |  | **0.32 (0.00367)** |  |  | **0.29 (0.00217)** |  |  |
| 69 | RH1_01695 3'(2'),5'-bisphosphate nucleotidase CysQ |  |  |  |  | **1.7 (0.02409)** |  |  |
| 70 | RH1_01700 Translation initiation factor IF-2 |  |  |  |  | **0.55 (0.03614)** |  |  |
| 71 | RH1_01705 preprotein translocase subunit SecA |  | **2.16 (0.03416)** |  |  | **1.94 (0.03929)** |  |  |
| 72 | RH1_01780 ATP-dependent protease ATPase subunit HslU |  | **2.09 (0.02568)** |  | **3.72 (0.01436)** |  |  |  |
| 73 | RH1_01783 Thioredoxin | **1.64 (0.02987)** |  |  |  |  |  |  |
| 74 | RH1_01793 H-NS histone family protein |  |  |  |  |  | **1.79 (0.0112)** | **0.60 (0.04802)** |
| 75 | RH1_01807 Acyl-CoA dehydrogenase |  |  |  |  |  |  | **0.56 (0.00988)** |
| 76 | RH1_01939 Dual-specificity RNA methyltransferase RlmN |  | **1.89 (0.00637)** |  |  | **1.61 (0.00825)** |  |  |
| 77 | RH1_01940 hypothetical protein | **0.64 (0.01762)** |  |  |  |  |  |  |
| 78 | RH1_01988 putative CtpA-like serine protease | **0,65 (0.00926)** |  |  |  |  |  |  |
| 79 | RH1_02008 Putative Inositol monophosphatase | **2.30 (0.03413)** | **5.44 (0.02487)** |  |  | **15.65 (0.0117)** | **6.15 (0.02754)** |  |
| 80 | RH1_02024 Nicotinamide-nucleotide amidohydrolase PncC |  |  |  | **1.98 (0.00395)** |  |  |  |
| 81 | RH1_02040 Nitrogen regulatory protein P-II |  |  |  |  | **1.58 (0.00944)** |  |  |
| 82 | RH1_02071 Ribosomal silencing factor RsfS |  | **0.47 (0.04164)** |  | **0.65 (0.01247)** |  |  |  |
| 83 | RH1_02091 Hypothetical protein |  | **0.29 (0.02458)** |  |  |  |  |  |
| 84 | RH1_02095 Hypothetical protein |  | **0.42 (0.00539)** |  |  |  | **0.52 (0.01797)** |  |
| 85 | RH1_02102 DNA-directed RNA polymerase subunit omega | **1.93 (0.00938)** |  |  |  |  |  |  |
| 86 | RH1_02115 Phosphoenolpyruvate carboxykinase [ATP] |  | **0.61 (0.01814)** |  |  |  |  |  |
| 87 | RH1_02117 Sensor histidine kinase YycG |  | **1.64 (0.00383)** |  |  |  |  |  |
| 88 | RH1_02130 DNA topoisomerase 4 subunit A |  | **0.25 (0.0157)** |  |  | **0.47 (0.05319)** |  |  |
| 89 | RH1_02142 DNA-directed RNA polymerase subunit beta' |  |  |  |  | **0.59 (0.05433)** |  |  |
| 90 | RH1_02145 30S ribosomal protein S7 | **1.6 (0.05575)** |  |  |  |  |  |  |
| 91 | RH1_02166 50S ribosomal protein L18 | **1.66 (0.00033)** |  |  |  |  |  |  |
| 92 | RH1_02167 30S ribosomal protein S5 |  |  |  |  |  | **0.63 (0.00113)** |  |
| 93 | RH1_02175 50S ribosomal protein L17 | **1.53 (0.01766)** |  |  |  |  |  |  |
| 94 | RH1_02186 Glutamine transport ATP-binding protein GlnQ |  |  |  |  |  |  | **0.64 (0.05429)** |
| 95 | RH1_02211 30S ribosomal protein S16 | **1.69 (0.0052)** |  |  |  |  |  |  |
| 96 | RH1_02229 30S ribosomal protein S15 | **1.53 (0.02953)** |  |  |  |  |  |  |
| 97 | RH1_02290 50S ribosomal protein L35 | **2.06 (0.01306)** |  |  |  |  |  |  |
| 98 | RH1_02318 Diaminopimelate decarboxylase |  | **1.68 (0.0000843)** | **1.62 (0.01866)** |  |  |  |  |
| 99 | RH1_02337 Alanine dehydrogenase |  |  |  |  |  |  | **1.59 (0.04924)** |
| 100 | RH1_02347 Putative ABC transporter substrate binding protein |  |  |  |  |  | **0.58 (0.05938)** |  |
| 101 | RH1_02355 Cytochrome c-552 |  | **0.58 (0.01067)** |  |  | **0.62 (0.00761)** | **0.63 (0.05182)** |  |
| 102 | RH1_02446 S-adenosylmethionine synthase |  | **0.65 (0.04882)** |  |  |  |  |  |
| 203 | RH1_02462 Glycine cleavage system H protein | **1.69 (0.03085)** |  |  |  |  |  |  |
| 104 | RH1_02464 Glycine dehydrogenase (decarboxylating) |  |  |  |  |  |  | **1.49 (0.00623)** |
| 105 | RH1_02467 Putative acyltransferase |  | **0.43 (0.01168)** |  |  |  |  |  |
| 106 | RH1_02495 Receptor family ligand binding region |  |  |  |  |  |  | **0.51 (0.02055)** |
| 107 | RH1_02508 Glutamine-binding periplasmic protein precurs |  |  |  |  |  | **1.51 (0.00465)** |  |
| 108 | RH1_02524 metal-dependent hydrolase |  |  | **1.58 (0.0025)** |  |  |  |  |
| 109 | RH1_02527 Putative Om channels superfamily, porina domain |  |  |  |  | **0.55 (0.03318)** |  |  |
| 110 | RH1_02573 Lysine--tRNA ligase |  | **1.75 (0.00575)** |  |  | **1.59 (0.00623)** |  |  |
| 111 | RH1_02575 Peroxiredoxin OsmC |  |  |  | **1.57 (0.04237)** |  | **0.44 (0.05587)** |  |
| 112 | RH1_02605 molybdopterin biosynthesis protein MoeB | **1.52 (0.01397)** |  |  |  |  |  |  |
| 113 | RH1_02638 Hypothetical protein | **1.51 (0.01941)** |  |  |  |  |  |  |
| 114 | RH1_02691 Glycine--tRNA ligase alpha subunit |  |  |  |  |  |  | **2.18 (0.04152)** |
| 115 | RH1_02726 Biopolymer transport protein ExbB |  |  |  |  |  | **1.65 (0.00396)** |  |
| 116 | RH1_02764 Membrane-bound lytic murein transglycosylase |  | **0.57 (0.03837)** |  |  |  |  |  |
| 117 | RH1_02897 Cell division protein FtsZ |  | **0.63 (0.02741)** |  |  | **0.63 (0.03798)** |  |  |
| 118 | RH1_02908 Putative Aldolase |  |  | **1.83 (0.03929)** |  |  |  |  |
| 119 | RH1_02932 Hypothetical protein |  |  | **1.63 (0.04126)** |  |  |  |  |
| 120 | RH1_02948 Threonine--tRNA ligase |  |  |  |  | **0.64 (0.03267)** |  |  |
| 121 | RH1_02953 Hypothetical protein | **2.25 (0.00261)** |  |  |  |  |  |  |
| 122 | RH1_02964 Octopine permease ATP-binding protein P |  |  |  |  |  | **0.50 (0.05254)** |  |
| 123 | RH1_02978 Multiple sugar-binding periplasmic receptor C |  | **0.40 (0.01575)** |  |  |  |  |  |
| 124 | RH1_03010 Ribosomal large subunit pseudouridine synthas |  |  |  | **0.25 (0.02472)** |  |  |  |
| 125 | RH1_03014 Oligoendopeptidase F, plasmid |  |  |  |  | **3.23 (0.04773)** |  | **1.85 (0.04046)** |
| 126 | RH1_03022 SCP-2 sterol transfer family protein | **1.52 (0.00319)** |  |  |  | **0.44 (0.02084)** |  |  |
| 127 | RH1_03027 RNA polymerase-binding transcription factor CarD | **2.04 (0.01066)** |  |  |  |  |  |  |
| 128 | RH1_03063 Histidinol dehydrogenase |  |  |  |  |  | **2.09 (0.05016)** |  |
| 129 | RH1_03129 Putative ABC transporter substrate binding protein |  | **0.63 (0.01741)** |  |  | **0.53 (0.00383)** |  | **0.60 (0.00779)** |
| 130 | RH1_03185 Hypothetical protein | **0.65 (0.01946)** |  |  |  |  |  |  |
| 131 | RH1_03216 ATP-dependent Clp protease proteolytic subuni | **1.55 (0.02494)** |  |  |  |  |  |  |
| 132 | RH1_03224 NAD-dependent dihydropyrimidine dehydrogenase | **0.60 (0.02809)** |  |  |  |  |  |  |
| 133 | RH1_03289 Vitamin B12-dependent ribonucleotide reductas |  |  |  |  | **0.51 (0.05498)** |  |  |
| 134 | RH1_03320 Replicative DNA helicase |  | **0.16 (0.00048)** |  | **0.25 (0.00015)** | **0.23 (0.02447)** |  | **0.36 (0.03279)** |
| 135 | RH1_03322 Dihydroorotase |  | **0.29 (0.0078)** |  |  |  |  |  |
| 136 | RH1_03326 Putative OmpA-MoB domain | **0.48 (0.00322)** |  |  |  |  |  |  |
| 137 | RH1_03332 Hypothetical protein |  |  |  |  |  | **4.1 (0.05527)** |  |
| 138 | RH1_03335 Salt stress induced outer membrane protein | **1.91 (0.00049)** |  |  |  |  |  |  |
| 139 | RH1_03360 Trigger factor |  |  |  |  |  |  | **0.60 (0.04394)** |
| 140 | RH1_03377 LOG family protein ORF6 in fasciation locus |  | **4.45 (0.00939)** |  |  |  |  |  |
| 141 | RH1_03378 LysM domain/BON superfamily protein |  | **2.30 (0.0000393)** |  |  | **2.08 (0.00976)** | **1.74 (0.05959)** |  |
| 142 | RH1_03380 Hypothetical protein |  |  |  |  |  |  | **0.41 (0.01106)** |
| 143 | RH1_03393 1-deoxy-D-xylulose 5-phosphate reductoisomera |  |  |  |  |  | **0.31 (0.01103)** | **2.71 (0.02507)** |
| 144 | RH1_03428 putative L,D-transpeptidase YbiS precursor |  |  |  | **0.61 (0.00643)** |  | **1.70 (0.02449)** |  |
| 145 | RH1_03437 Enamine/imine deaminase | **1.9 (0.04599)** |  |  |  |  |  |  |
| 146 | RH2_00080 2,4-dichlorophenol 6-monooxygenase |  |  | **0.28 (0.02121)** |  |  |  |  |
| 147 | RH2_00188 Long-chain-fatty-acid--CoA ligase FadD13 |  |  |  |  | **2.73 (0.04203)** |  |  |
| 148 | RH2_00221 Homocysteine S-methyltransferase |  | **1.72 (0.03594)** |  |  | **1.87 (0.05314)** | **2.44 (0.05522)** |  |
| 149 | RH2_00229 Universal stress protein family protein |  | **0.55 (0.02681)** |  |  |  |  |  |
| 150 | RH2_00239 Outer membrane protein TolC precursor |  |  |  |  |  |  | **0.07 (0.00028)** |
| 151 | RH2_00279 Acetyl-coenzyme A synthetase |  |  |  |  | **0.66 (0.03987)** |  |  |
| 152 | RH2_00344 Periplasmic dipeptide transport protein precu |  |  |  |  | **0.55 (0.02889)** |  | **0.62 (0.02113)** |
| 153 | RH2_00348 Glutathione import ATP-binding protein GsiA |  |  | **1.65 (0.01671)** |  | **0.25 (0.01434)** |  |  |
| 154 | RH2_00427 50S ribosomal protein L27 |  |  |  |  | **1.57 (0.02403)** |  |  |
| 155 | RH2_00433 p-aminobenzoyl-glutamate hydrolase subunit B |  | **0.41 (0.00769)** |  |  |  |  |  |
| 156 | RH2_00457 Acetolactate synthase isozyme 2 large subunit |  | **0.32 (0.01286)** |  |  |  |  |  |
| 157 | RH2_00458 Leu/Ile/Val/Thr-binding protein precursor |  |  |  | **0.59 (0.04872)** |  |  |  |
| 158 | RH2_00473 Bacterial extracellular solute-binding protei | **0.62 (0.02076)** |  |  |  |  |  |  |
| 159 | RH3_00030 Ribose import ATP-binding protein RbsA |  |  |  | **1.65 (0.04645)** |  |  |  |
| 160 | RH3_00056 D-ribose-binding periplasmic protein precursor |  | **1.61 (0.00065)** |  |  |  |  |  |
| 161 | RH3_00135 Spermidine/putrescine-binding periplasmic pro | **0.57 (0.00811)** |  |  |  |  |  |  |
| 162 | RH3_00139 Sulfate adenylyltransferase subunit 2 |  |  |  |  |  |  | **0.58 (0.03967)** |
| 163 | RH3_00157 Alginate biosynthesis protein AlgA |  | **0.65 (0.04723)** |  |  |  | **0.60 (0.01828)** |  |
| 164 | RH3_00200 Oligopeptide transport ATP-binding protein Op |  | **0.18 (0.00728)** |  |  | **0.18 (0.0135)** |  |  |
| 165 | RH3_00272 Purine nucleoside permease (NUP) | **0.46 (0.00233)** | **0.38 (0.01579)** |  |  |  |  |  |
| 166 | RH3_00312 Chromosome partitioning protein ParA |  | **1.51 (0.05058)** |  |  | **0.55 (0.01643)** |  |  |
| 167 | RH3_00340 Glycosyl transferases group 1 | **1.71 (0.02567)** |  |  |  |  |  |  |
| 168 | RH5_00089 Hypothetical protein |  |  |  |  | **0.53 (0.04862)** |  | **0.41 (0.00631)** |
| 169 | RH5_00094 GDP-mannose-dependent alpha-mannosyltransfera | **0.48 (0.04725)** |  |  |  |  |  |  |
| 170 | RH5_00103 dTDP-4-dehydrorhamnose reductase |  |  |  | **0.51 (0.04927)** |  |  |  |
| 171 | RH5_00110 Hypothetical protein | **1.56 (0.02866)** |  |  |  |  |  |  |
