# Supplementary material for: Bacterial Survival under Extreme UV Radiation: A Comparative Proteomics Study of Rhodobacter sp., Isolated from High Altitude Wetlands in Chile
Source: Front Microbiol. 2017 Jun 26;8:1173. doi: 10.3389/fmicb.2017.01173 (PMC5483449; doi:10.3389/fmicb.2017.01173)
Supplement: Supplementary file 4 [file Table_4.DOCX]

**Table S4.** List of hypothetical proteins and re-annotated hypothetical proteins with their predictive putative functions.

| **HYPOTHETICAL PROTEINS** | **REANNOTATION** | **QUERY COVER** | **E VALUE** | **IDENTITY** | **ACCESSION NUMBER** | **FUNCTIONAL CATEGORIES** |
| --- | --- | --- | --- | --- | --- | --- |
| **RH1_00146** | Hypothetical protein | - | - | - | - | HYPOTHETICAL PROTEIN |
| **RH1_00571** | Hypothetical protein | - | - | - | - | HYPOTHETICAL PROTEIN |
| **RH1_00660** | Cell division MukB domain | 100% | 5E-99 | 74% | WP_011908947.1 | CELL DIVISION |
| **RH1_00731** | Putative lipoprotein | 100% | 4e-37 | 60% | EEW26478.1 | ENERGY PRODUCTION |
| **RH1_00945** | Hypothetical protein | - | - | - | - | HYPOTHETICAL PROTEIN |
| **RH1_00947** | Salt stress induced outer membrane protein | 100% | 7E-144 | 69% | WP_009561769.1 | CELL ENVELOPE BIOGENESIS |
| **RH1_01940** | Hypothetical protein | - | - | - | - | HYPOTHETICAL PROTEIN |
| **RH1_02008** | Inositol monophosphatase | 98% | 3E-46 | 89% | WP_002722116.1 | ANTIOXIDANT |
| **RH1_02091** | Hypothetical protein | - | - | - | - | HYPOTHETICAL PROTEIN |
| **RH1_02095** | Hypothetical protein | - | - | - | - | HYPOTHETICAL PROTEIN |
| **RH1_02347** | Hypothetical protein | - | - | - | - | HYPOTHETICAL PROTEIN |
| **RH1_02527** | Hypothetical protein | - | - | - | - | HYPOTHETICAL PROTEIN |
| **RH1_02638** | Isoquinoline 1- oxidoreductase | 100% | 7E-102 | 77% | WP_041670365.1 | ENERGY PRODUCTION |
| **RH1_02908** | Aldolase | 100% | 5E-52 | 78% | WP_002719280.1 | ENERGY PRODUCTION |
| **RH1_02932** | Hypothetical protein | - | - | - | - | HYPOTHETICAL PROTEIN |
| **RH1_02953** | Hypothetical protein | - | - | - | - | HYPOTHETICAL PROTEIN |
| **RH1_03129** | ABC transporter substrate binding protein | 100% | 0 | 88% | WP_049763956.1 | CELL TRANSPORT |
| **RH1_03185** | Hypothetical protein | - | - | - | - | HYPOTHETICAL PROTEIN |
| **RH1_03326** | Membrane protein/OmpA-MoB domain | 99% | 4E-134 | 43% | WP_041669119.1 | CELL ENVELOPE BIOGENESIS |
| **RH1_03332** | Hypothetical protein | - | - | - | - | HYPOTHETICAL PROTEIN |
| **RH1_03335** | Sulfite reductase | 100% | 2 E-56 | 86% | WP_011840456.1 | ENERGY PRODUCTION |
| **RH1_03380** | Hypothetical protein | - | - | - | - | HYPOTHETICAL PROTEIN |
| **RH5_00089** | Hypothetical protein | - | - | - | - | HYPOTHETICAL PROTEIN |
| **RH1_01065** | Peptidase S15 | 100% | 0 | 70% | WP_008028283.1 | CHAPERONES & PROTEASES |
| **RH5_00110** | Hypothetical protein | - | - | - | - | HYPOTHETICAL PROTEIN |
