# Supplementary figures and images for: Bacterial Survival under Extreme UV Radiation: A Comparative Proteomics Study of Rhodobacter sp., Isolated from High Altitude Wetlands in Chile
Source: Front Microbiol. 2017 Jun 26;8:1173. doi: 10.3389/fmicb.2017.01173 (PMC5483449; doi:10.3389/fmicb.2017.01173)

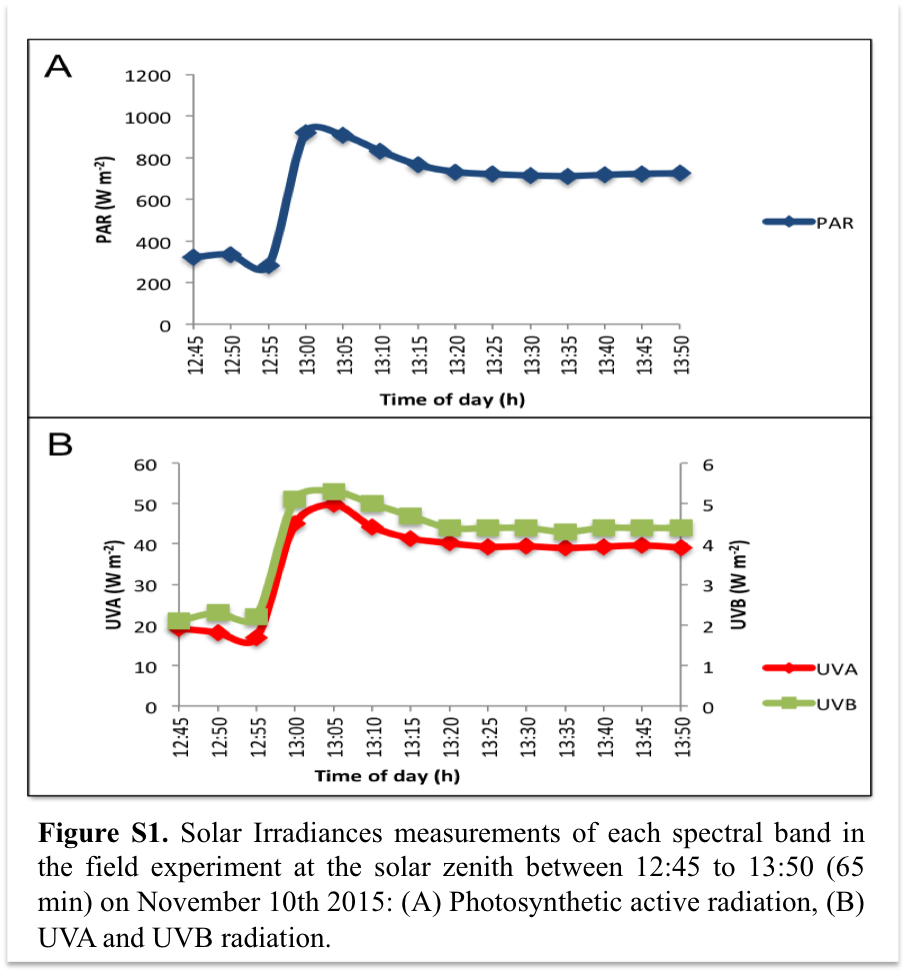

Supplement: Supplementary file 7 [file Image_1.JPEG]

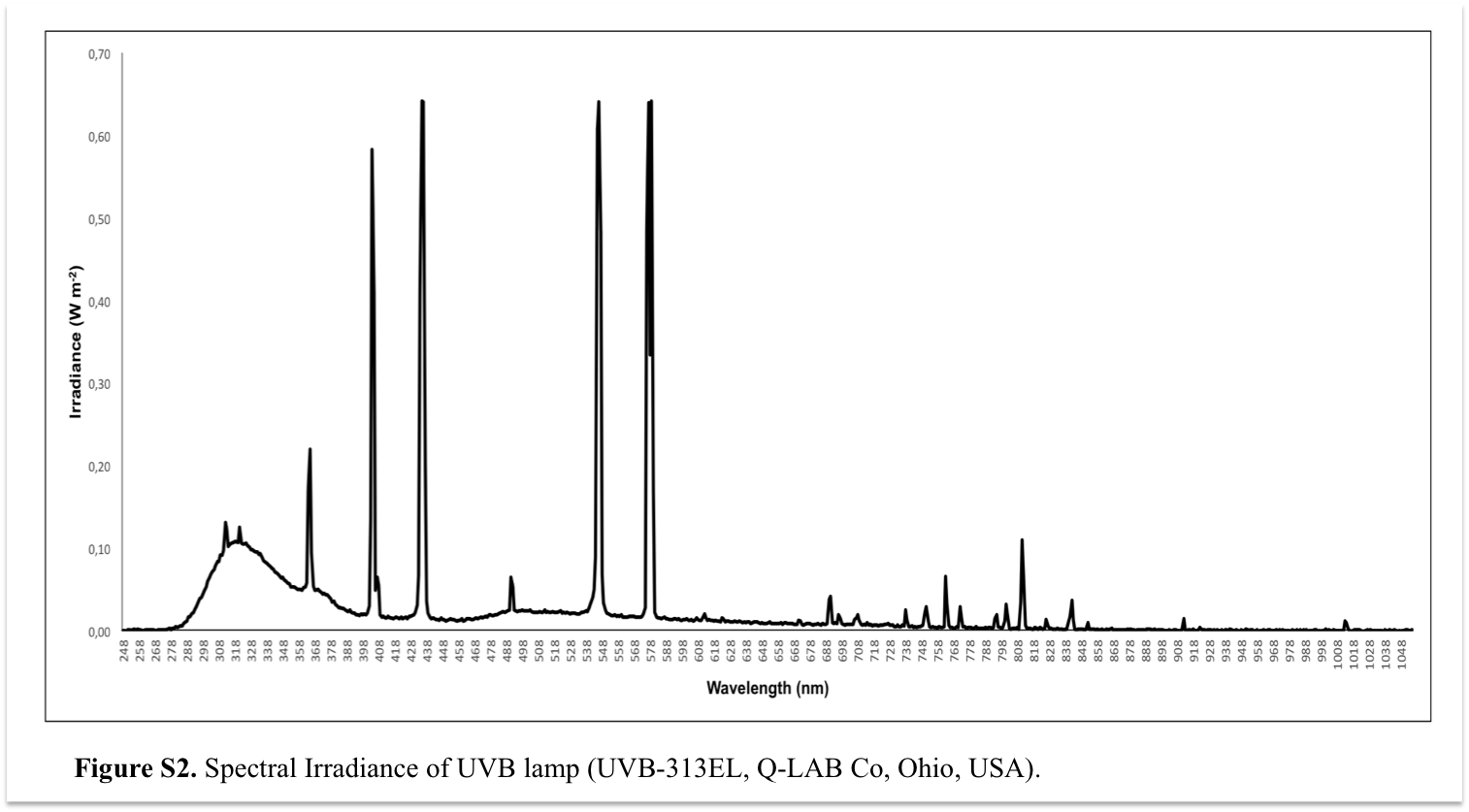

Supplement: Supplementary file 8 [file Image_2.png]

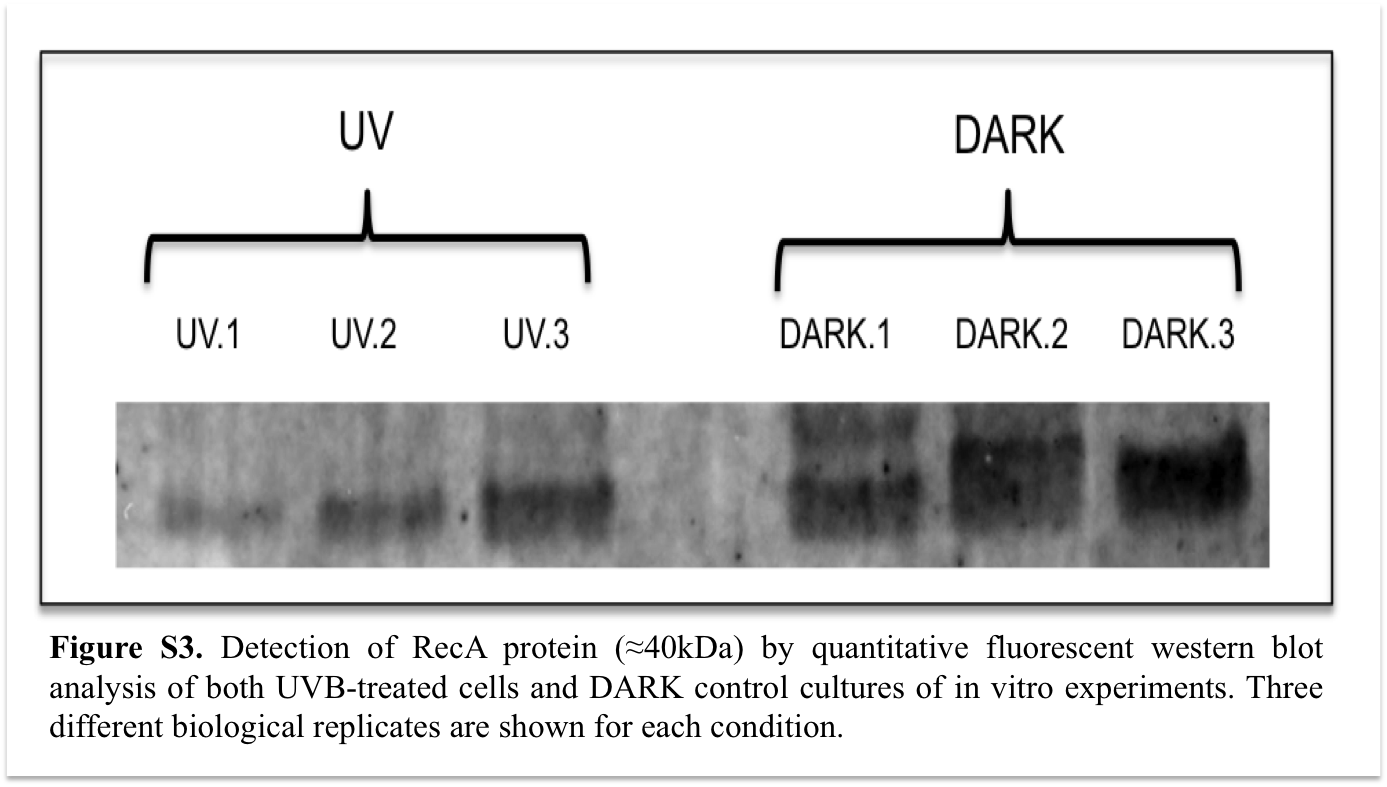

Supplement: Supplementary file 9 [file Image_3.JPEG]
